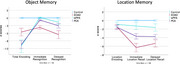# Memory impairment across the atypical AD syndromic spectrum

**DOI:** 10.1002/alz70857_099054

**Published:** 2025-12-24

**Authors:** Deepti Putcha, Kanella Basilion, Yuta Katsumi, Brad Dickerson

**Affiliations:** ^1^ Frontotemporal Disorders Unit, Department of Neurology, Massachusetts General Hospital and Harvard Medical School, Boston, MA, USA; ^2^ Massachusetts General Hospital, Boston, MA, USA; ^3^ Department of Neurology, Massachusetts General Hospital and Harvard Medical School, Boston, MA, USA

## Abstract

**Background:**

The diagnostic criteria for atypical syndromes of Alzheimer's disease (AD) specify that episodic memory is relatively preserved at initial stages and may develop as the disease progresses. Memory deficits have been reported in posterior cortical atrophy (PCA), and logopenic variant primary progressive aphasia (lvPPA), despite these groups being referred to as “non‐amnestic”. The shared and dissociable patterns of memory impairment across atypical syndromes have not yet been clearly delineated.

**Method:**

We tested 16 early‐onset (EOAD), 9 lvPPA, 21 PCA, and 29 cognitively normal (CN) participants with a novel object‐location memory test (OLMT) designed to foster deep learning and interrogate associative memory between objects and locations without lexical retrieval demands. Analysis of variance (ANOVA) and post‐hoc *t*‐tests were conducted to characterize between group performance. General linear models interrogated the association of atrophy in the default mode network with different stages of memory.

**Result:**

All atypical AD variants demonstrated impaired encoding over three learning trials compared to CNs, with a positive learning curve. EOAD demonstrated greater storage loss compared to the other groups at 3‐minute delayed recognition (vs. PCA: *t* = 3.2, *p* = 0.002; vs. lvPPA: *t* = 3.1, *p* = 0.005) but comparable levels of impairment at 30‐minute delayed recognition (EOAD: z=‐4.4 ± 6.1; PCA: z=‐2.2 ± 3.6; lvPPA: z=‐1.2 ± 2.1). While the lvPPA group was able to effectively associate locations with objects and retain this association over time, other variants struggled with both encoding (EOAD: z=‐4.4 ± 6.1; PCA: z=‐2.2 ± 3.6) and retention (EOAD: z=‐4.4 ± 6.1; PCA: z=‐2.2 ± 3.6) of spatial locations over time. Medial temporal lobe atrophy was uniquely associated with object‐location associative binding and storage over time, but not object encoding alone.

**Conclusion:**

All atypical AD syndromes demonstrated poor encoding and storage loss, adding to the characterization of memory impairment in atypical “non‐amnestic” AD. EOAD participants demonstrated faster storage loss compared to the other variants, potentially reflecting multidomain impairment. While PCA and lvPPA performed comparably on object memory, these groups were differentiated by location memory binding. Understanding the varied presentation of memory deficits in atypical syndromes can help inform accurate diagnosis and cognitive skills training to cope with neurodegenerative decline.